# Assessing Medical Students’ Understanding of Do-Not-Resuscitate (DNR) Orders

**DOI:** 10.7759/cureus.69132

**Published:** 2024-09-10

**Authors:** Omar Basubrin

**Affiliations:** 1 Faculty of Medicine, Umm Al-Qura University, Makkah, SAU

**Keywords:** physician's decision, practice, attitude, dnr, medical students, saudi arabia, knowledge

## Abstract

Objective: The evolution of medical directives surrounding cardiopulmonary resuscitation (CPR) has led to the prominence of do-not-resuscitate (DNR) orders. These orders aim to respect patient autonomy and ensure dignified end-of-life care. While regulations in the United States emphasize patient choice and comprehensive discussions, Saudi Arabia's approach to DNR orders integrates Islamic principles alongside expert medical judgment. This study aimed to assess the awareness and comprehension of DNR orders among medical students at Umm Al-Qura University, Makkah, Saudi Arabia.

Methods: This study employed a cross-sectional design at Umm Al-Qura University in 2023. Convenience sampling yielded a sample of 145 participants, encompassing medical students from all years (first-sixth) and medical interns. A self-administered online questionnaire assessed participants' knowledge, beliefs, and attitudes regarding DNR orders. The questionnaire specifically addressed their familiarity with DNR orders, their understanding of the Islamic perspective on DNR, and the factors influencing their attitudes toward DNR.

Results: The majority of respondents (62%) were familiar with the term "DNR," but only 52% correctly defined it. Social media was the primary source of information about DNR for most respondents (56%). A significant portion of respondents (60.7%) agreed with the concept of DNR, and 54% had personal experience with the term. Patient dignity (54%), religious concerns (66.7%), and legal concerns (60.7%) were identified as important factors in DNR decisions. A majority of respondents (67%) agreed that it is acceptable to discontinue life support for DNR patients, and 49.3% believed that organ donation should be encouraged among patient families. The study found that as medical students progressed through their training (from pre-clinical to clinical years and internship), their acceptance and understanding of DNR orders generally increased.

Conclusion: The study found that medical students possess some basic familiarity with DNR orders, but there is considerable room for advancement in their comprehension and acceptance of the topic. Addressing these knowledge gaps may lead to improved patient outcomes. The findings also underscore the need to equip future physicians with a comprehensive understanding of DNR orders, their ethical ramifications, and effective communication strategies for engaging with patients and their families.

## Introduction

In recent years, allowing natural death has gained popularity over the traditional do-not-resuscitate (DNR) order, though the latter remains common in many hospitals [1]. A DNR order instructs healthcare providers to refrain from cardiopulmonary resuscitation (CPR) during cardiac or respiratory arrest, without therapeutic recommendations. DNR adoption is widespread and involves significant medical-legal considerations, often leading to disputes [2]. In the United States, evolving DNR regulations since the 1980s emphasize patient autonomy, requiring comprehensive discussions between clinicians and patients or their representatives prior to drafting a DNR order, respecting their choice regardless of acceptance or rejection [3].

In Saudi Arabia, DNR regulations are influenced by Islamic principles, with fatwas (religious rulings issued by qualified Islamic scholars) holding authority over policy and law [4]. According to Fatwa number 12086 issued in 1988/1409 AH (Anno Hegirae in Latin or the year of the Hijra), a DNR order will be issued if three competent experts deem further resuscitation attempts futile, regardless of the patient's or family's wishes [5]. The regional DNR standards aim to provide necessary treatments and interventions, except CPR, while ensuring patients die with respect and dignity [6].

CPR is an emergency procedure used to revive individuals experiencing cardiac arrest [7]. However, only a small percentage fully recover, with many requiring further medical care and facing risks of permanent damage [8]. In such cases, DNR orders serve as directives indicating that no CPR measures should be taken. These orders, issued by physicians, may be recommended for patients with poor prognoses or unlikely survival with CPR, aiming to prevent prolonged suffering. Patients can also opt for a DNR order in advance. Importantly, DNR orders include CPR instructions but exclude food, medication, and pain relief [9].

In Saudi Arabia, the decision to issue a DNR order rests with three qualified physicians based on their assessment of medical futility. While the patient's direct involvement is ideal, family members are actively engaged in the discussion, particularly in cases where the patient may be unable to make decisions due to their medical condition. Due to patients' and families' limited knowledge, medical professionals typically make DNR decisions [10]. However, there is a lack of research on the perceptions of DNR orders among medical students and residents in Saudi Arabia. This study aims to examine the attitudes regarding and understanding of DNR orders among medical students and interns at Umm Al-Qura University College of Medicine, Makkah, Saudi Arabia, addressing this gap.

## Materials and methods

Research design

This cross-sectional study was conducted without any intervention using a self-administered online questionnaire in 2023 at Umm Al-Qura University in the Kingdom of Saudi Arabia.

Sampling

Convenience sampling was utilized for participant recruitment in this study. Convenience sampling involves selecting participants based on their accessibility and availability to the researcher. In this study, individuals who voluntarily filled out the distributed questionnaire were included as participants. The study sample comprised 145 medical students from first year to sixth year and medical interns. The sample population included 44% males and 56% females. A copy of the questionnaire has been provided in the appendices.

Data collection and analysis

Data was collected using a customized self-administered online questionnaire. Descriptive statistics were used to assess medical students' and interns' familiarity with and beliefs about DNR orders, as well as their understanding of the Islamic perspective on DNR orders. Additionally, the study explored medical students' attitudes toward DNR orders and the factors influencing these attitudes.

## Results

Demographic analysis

All participants provided informed consent to participate in the study, and responses were collected using the questionnaire. The following breakdown of respondents by year of medical education was observed: 10% (15) were in their first year, 15.3% (22) were in their second year, 11% (16) were in their third year, 10.65% (15) were in their fourth or fifth year, and 31.3% (45) were in their sixth year of medical education. Additionally, 11% (16) of the respondents were interns. Regarding gender, 56% (81) of the respondents were female and 44% (64) were male. Marital status data indicated that 89.3% (129) of the students were single, while 10.7% (16) were married (Table 1).

**Table 1:** Demographic analysis The data has been represented as N followed by % in brackets. Statistical significance was set at a p-value less than 0.05

Demographic	Characteristic	N (%)
Consent	Agree	145 (100)
	Not agree	0
Medical year	Firstyear	15 (10)
	Second year	22 (15.3)
	Third Year	16 (11)
	Fourth Year	15 (10.65)
	Fifth Year	15 (10.65)
	Sixth Year	45 (31.3)
	Interns	16 (11)
Gender	Female	81 (56)
	Male	64 (44)
Social status	Single	129 (89.3)
	Married	16 (10.7)

Knowledge about DNR orders

A majority 62% (90) of the respondents were familiar with the term "DNR." However, only 52% (75) of respondents correctly defined DNR. A significant portion 56% (81) of respondents first encountered the term DNR through social media, highlighting its prevalence in online spaces. While 44% (64) of respondents first learned about DNR from faculty staff, indicating that they had no prior knowledge of the term before entering university. Interestingly, 60.7% (88) of the total respondents agreed with the concept of DNR, and 54% (78) had personal experience with the term. Notably, 86% of respondents acknowledged the existence of a fatwa (religious decree) regarding DNR (Table 2).

**Table 2:** Knowledge about DNR orders The data has been represented as N followed by % in brackets. Statistical significance was set at a p-value less than 0.05

Question	Answer	N (%)
Are you familiar with the term “do-not-resuscitate (DNR) order”?	Yes	90 (62)
	No	46 (32)
Definition of a DNR order	Medical order written by a doctor	75 (52)
	An order or prior instructions	24 (16.7)
	Both are right	26 (18)
	Other	19 (13.3)
Where did you hear about DNR orders?	Social media	81 (56)
	University staff	64 (44)
Are you in agreement with DNR orders?	Yes	88 (60.7)
	No	57 (39.3)
Do you have personal experience with a DNR order?	Yes	78 (54)
	No	57 (39.3)
	More than one	10 (6.7)
Who should give DNR orders?	Doctors	78 (54)
	Family members	40 (27.3)
	Nurses	4 (2.7)
	Doctors and family members	4 (2.7)
	Nurses and doctors	4 (2.7)
	Others	3 (2)
	No one	12 (8.7)
Is there a fatwa regarding DNR orders?	Yes	125 (86)
	No	9 (6)
	Not sure	11 (8)

Factors contributing to DNR decisions

Data analysis revealed that patient dignity was considered an important factor in DNR decisions by 54% (78) of respondents, while a larger proportion 66.7% (97) viewed religious concerns as an important factor as well. Additionally, 60.7% (88) of respondents believed that legal concerns played a crucial role in DNR decision-making. Furthermore, 64% (93) of respondents acknowledged the risk of a vegetative state as a significant factor in DNR decisions, while 66.7% (97) emphasized the importance of efficient resource utilization and cost reduction in DNR considerations (Table 3).

**Table 3:** Factors affecting DNR decisions The data has been represented as N followed by % in brackets. Statistical significance was set at a p-value less than 0.05

Factor	Response	N (%)
Patient dignity	Yes	78 (54)
	No	57 (39.3)
	Not Sure	10 (6.7)
Religious concern	Yes	97 (66.7)
	No	48 (33.3)
Legal concerns	Yes	88 (60.7)
	No	57 (39.3)
Risk of vegetative state	Yes	93 (64)
	No	52 (36)
Limited availability of ICU beds	Yes	82 (56.7)
	No	63 (43.3)
Efficient use of medical resources and cost reduction	Yes	97 (66.7)
	No	48 (33.3)

Attitudes regarding DNR orders

The survey results revealed that 46.7% (68) of respondents believe it is acceptable to proceed cautiously with investigations and therapies for patients for whom DNR orders are issued. A majority of 60.7% (88) of those polled agree that it is acceptable to discontinue life support for such patients. Additionally, 49.3% (71) of respondents believe that organ donation should be encouraged among patient families. Notably, 63.3% (92) of respondents found discussions about DNR orders to be stressful, while 60% (87) of respondents agreed on the importance of DNR orders (Table 4).

**Table 4:** Attitudes toward DNR orders 1=strongly agree, 2=agree, 3=neutral, 4=Disagree, 5=Strongly disagree The data has been represented as N followed by % in brackets. Statistical significance was set at a p-value less than 0.05

Belief	Response	N (%)
Patients who are subject to do-not-resuscitate (DNR) orders are allowed to have more cautious examinations and treatments.	1	68 (46.7)
	2	35 (24)
	3	13 (9.3)
	4	16 (10.7)
	5	13 (9.3)
Patients who are subject to DNR orders might have their life-sustaining therapy withdrawn.	1	88 (60.7)
	2	25 (17.3)
	3	13 (9.3)
	4	9 (6)
	5	10 (6.7)
It's important to persuade patients subject to DNR orders and/or their families to talk about organ donation.	1	71 (49.3)
	2	42 (28.7)
	3	13 (9.3)
	4	10 (6.7)
	5	9 (6)
Discussions about DNR orders are stressful.	1	92 (63.3)
	2	7 (4.7)
	3	15 (10)
	4	16 (11.3)
	5	15 (10.7)
DNR orders are important.	1	87 (60)
	2	7 (4.7)
	3	10 (6.7)
	4	16 (11.3)
	5	25 (17.3)

Moreover, a one-way analysis of variance (ANOVA) test revealed that the attitude and knowledge about DNR orders differ among medical year students and interns. The participants were divided into three groups: group 01 included students from years two, three, and four; group 02 included interns; and group 03 included students from clinical years five and six. The findings indicated that the difference in means within groups was larger than the difference between groups. These results suggest that with progression in academic years, the acceptance level and knowledge about DNR orders are enhanced, implying that the knowledge about DNR orders improves in higher academic years. Therefore, it can be inferred that there exists a difference in attitude between those who are knowledgeable about DNR orders compared to those who have no knowledge about DNR orders. The findings of the ANOVA test are given in Table 5.

**Table 5:** Analysis of variance (ANOVA) test results df: degrees of freedom Statistical significance was set at a p-value less than 0.05

	Sum of squares	df	Mean square	F	Significance
Between groups	0.082	1	0.082	0.046	0.831
Within groups	267.391	148	1.807		
Total	267.473	149			

## Discussion

This study aimed to assess the awareness and comprehension of DNR orders among medical students at Umm Al-Qura University. The findings highlight several critical issues that warrant attention in the training of future healthcare professionals.

Implications for medical education

While most medical students supported the concept of withdrawing life support in DNR situations, their grasp of the specific legal and regulatory framework appears limited. Current Saudi Arabian guidelines stipulate initial CPR until DNR status is confirmed, and allow for withholding or withdrawing life support only after a DNR order has been assigned, and only under specific circumstances of medical futility or excessive patient burden, as determined by the attending physician [11]. This highlights a need for targeted education to ensure medical students fully understand the complexities of DNR decision-making.

The research results also revealed a diversity of opinions among medical students regarding DNR orders. Students expressed varying sentiments about engaging in difficult conversations with patients and their loved ones about end-of-life care. These divergent perspectives emphasize the necessity of incorporating instruction on the emotional and communicative aspects of end-of-life care into medical education.

The study's findings could serve as a catalyst for Umm Al-Qura University to refine its teaching methodologies and maintain its competitive edge in the academic landscape. Integrating comprehensive DNR instruction into the medical curriculum would better equip medical students to navigate these challenging conversations with patients and their families, ultimately benefiting all parties involved.

To address the identified knowledge gaps and foster a deeper, more nuanced understanding of DNR orders, it is recommended that Umm Al-Qura University should introduce specialized training modules or seminars. These modules could incorporate discussions on legal frameworks, ethical dilemmas, and effective communication strategies, empowering the next generation of healthcare professionals to engage confidently in these complex conversations.

Comparison with the existing literature

In a similar study conducted in Jeddah, Saudi Arabia, [2], 82.8% (352) of 425 medical students were aware of DNR orders; however, only 28.7% (122) of the participants chose the correct definition. The study concluded that there was a significant lack of understanding of DNR orders among medical students in Saudi Arabia, sharing similarities with our findings and highlighting the lack of ethical education regarding DNR orders and their policies.

In a study conducted at King Abdulaziz University Hospital (KAUH) in Jeddah [3], it was found that 73.2% (314) of the 429 medical students surveyed were aware of DNR orders. Alarmingly, 58.3% (250) of these students had not received any formal lecture or session on DNR. The study revealed varying levels of knowledge about DNR across different levels of medical training, with interns demonstrating greater knowledge compared to second-year medical students.

On the other hand, in another study conducted at Universiti Sains Malaysia, George Town, Malaysia, [12], 84.4% (211) of participants were aware of DNR orders, with 38% (95) citing lectures or formal discussions as their main source of information, followed by social media and the internet 27.2% (68). This contrasts with our results. The students in the study demonstrated good awareness of DNR orders despite belonging to a multicultural community, emphasizing the importance of lectures on DNR orders, which are currently lacking in Saudi Arabia.

Implications for end-of-life care

Every patient should engage in a discussion with their healthcare provider regarding their DNR and advanced directive status. A primary objective of this discussion is to dispel the misconception that a DNR order automatically implies a poor quality of life. It is crucial to reassure patients' loved ones that every effort will be made to alleviate their loved one's suffering in any feasible manner. Educating future physicians and other medical professionals about DNR is as essential as educating the general public. By equipping medical students and healthcare personnel with a comprehensive understanding of the ethical principles underlying end-of-life care through DNR education, they may make more informed and ethical decisions in the implementation of DNR directives. Additionally, DNR education can foster enhanced communication between patients, their families, and their clinicians. DNR training can also improve other aspects of hospice care provided to patients nearing the end of their lives.

Directions for future research

This study serves as a foundation for further research endeavors aimed at understanding the evolving context in which medical students' attitudes toward end-of-life care are shaped. Several areas warrant further investigation, including the long-term benefits of educational programs and the influence of culture on individuals' perceptions of DNR directives.

Overall, the findings of this research emphasize the need to address the knowledge and attitude gaps that have been identified in the healthcare field. There is a critical demand for compassionate and knowledgeable medical professionals capable of navigating the complexities of end-of-life care. Umm Al-Qura University can play a pivotal role in meeting this demand by developing and implementing a range of educational programs and activities.

Limitations of this study

The cross-sectional design of the study limited our ability to investigate causal relationships between variables. Additionally, the use of convenience sampling and online survey administration may have restricted the generalizability of the findings, as a significant portion of the target population might not have internet access. Although a pilot study was conducted to evaluate the external validity of the research instrument, test-retest or inter-rater reliability testing was not performed.

## Conclusions

This study offers valuable insights into the attitudes and understanding of DNR orders among medical students at Umm Al-Qura University. While medical students possess some basic familiarity with DNR orders, there is considerable room for advancement in their comprehension and acceptance of the topic. Given the critical role of DNR decisions in patient care and the ethical considerations involved, addressing these knowledge gaps can lead to improved patient outcomes by fostering informed decision-making, reducing conflicts, and ensuring patient-centered, ethically sound end-of-life care. The findings also underscore the need to equip future physicians with a comprehensive understanding of DNR orders, their ethical ramifications, and effective communication strategies for engaging with patients and their families. As we endeavor to shape the next generation of medical professionals, it is paramount to instill in them the importance of prioritizing patient well-being and making ethical choices during end-of-life care.

## Appendices

Section A

This section contained general questions asked to all participants (Table 6).

**Table 6:** Section A M: Male, F: Female

Question	Options
Level of Study	1-6 + Intern
Gender	M, F
Social Status	Single, Married
Are you familiar with the term “do-not-resuscitate order”	Yes, No

Section B

This section had questions that were asked if participants answered YES to the last question in Table 6 (Table 7).

**Table 7:** Section B

Question	Options					
What is a do-not-resuscitate (DNR) order?	Medical order written by a doctor. It instructs healthcare providers not to do cardiopulmonary resuscitation (CPR) if a patient's breathing stops or if the patient's heart stops beating	The order or prior instructions issued by the doctor or the person authorized to prevent healthcare providers from doing cardiopulmonary resuscitation (CPR) if a patient’s breathing stops or if the patient’s heart stops beating.	Both are right	Other		
Where did you hear about it?	Social Media	Internet	Relative	Encounter with healthcare	University staff	Other
Are you in agreement with DNR orders?	Yes, No					
Do you have a personal experience with DNR orders? More than one	No	A friend	A relative	A patient		
Who should give DNR orders? More than one	Doctors	Nurses	Family members	No one		
Who should be aware of a standing DNR order for a patient? More than one	Patient	Siblings	Parents	Spouse	No one	
Is there a fatwa regarding DNR orders?	Yes, No					

Section C

If answered No to the last question in Section A then the definition “Medical order written by a doctor. It instructs healthcare providers not to do cardiopulmonary resuscitation (CPR) if a patient's breathing stops or if the patient's heart stops beating” will be displayed followed by the following questions in Table 8.

**Table 8:** Section C

Question	Options					
What do you understand from the definition? More than one	DNR means withholding all treatment	Supportive measures are performed in the patient with DNR orders (feeding, fluids, oxygen)	DNR means "no care"	DNR means "do not treat or investigate"	Cardiopulmonary resuscitation (CPR) is not done in patients with DNR orders	Did not get the definition
Are you in agreement with DNR orders?	Yes, No					
Who should give DNR orders? More than one	Doctors	Nurses	Family members	No one		
Who should be aware of a standing DNR order for a patient? More than one	Patient	Siblings	Parents	Spouse	No one	
Is there a fatwa regarding DNR?	Yes, No					

Section D

All participants were then asked, “What factors should play a role in the decision to issue a DNR order?" (Table 9).

**Table 9:** Section D

Question	Options	
Patient Dignity	Yes, No	
Religious Concerns	Yes, No	
Legal concerns	Yes, No	
Risk of Vegetative State	Yes, No	
Limited availability of ICU beds	Yes, No	
Efficient use of medical resources and cost reduction	Yes, No	

Section E

To assess attitudes toward DNR, all participants were asked, “To what extent do you agree with the following statements?” (Table 10).

**Table 10:** Section E

Question	Options
It is acceptable to be conservative in investigations and treatments with patients who are labeled as do-not-resuscitate (DNR) patients.	Scale 1-5
It is acceptable to withdraw life-sustaining treatment from DNR-labeled patients.	Scale 1-5
The discussion of organ donation with DNR-labeled patients and/or their families should be encouraged.	Scale 1-5
It is best that patients are not made aware of their DNR status.	Scale 1-5
Discussions about DNR orders are stressful.	Scale 1-5
Do you think that a DNR order is important?	Scale 1-5
